# Factors associated with adaptation level in the older adult residential care facilities: a path analysis

**DOI:** 10.3389/fpubh.2023.1085399

**Published:** 2023-09-28

**Authors:** Di Zhao, Meilan Niu, Shanfeng Zhang, Yan Shi, Lin Zhou, Yuxia Song, Rui Ma, Peng Wang

**Affiliations:** ^1^Department of Community Care, School of Nursing and Health, Zhengzhou University, Zhengzhou, Henan, China; ^2^Department of Pharmacology, Medical School of Huanghe Science and Technology University, Zhengzhou, Henan, China; ^3^Experimental Center for Basic Medicine, Biochemistry and Molecular Biology, Zhengzhou University, Zhengzhou, Henan, China; ^4^Henan Electric Power Hospital, Zhengzhou, Henan, China; ^5^College of Physical Education, Zhengzhou University, Zhengzhou, Henan, China

**Keywords:** social ecosystem theory, the older adult, adaptation, a path analysis, residential care facilities

## Abstract

**Background:**

It has become very common for older adults to relocate to residential care facilities. Yet whether older adults adapt to life in a long-term care residential facility through perception, reflection, and conscious behavioral choices is a challenging social issue. Previous research has shown that adaptation is influenced by physical, mental, psychological, social systems, and other debris factors. However, existing knowledge is often based on unidirectional relationships between these factors and adaptation. Few studies have formally examined bivariate relationships between these factors, and the influence of adaptation between these factors internally remains unclear. Therefore, there is a need to examine the structural causality of adaptation in residential care facilities influenced by a combination of physical, emotional, social and psychological factors, life satisfaction, and social support.

**Methods:**

The present cross-sectional study recruited older adults from three residential care facilities in Henan province, China, through convenience sampling. The Chinese Nursing Home Adjustment Scale (NHAS), Geriatric Depression Scale-15 (GDS-15) and Social Support Scale (SSRS), Satisfaction with Life Scale (SWLS), and Barthel Index were employed to measure the older adult’ adjustment level, depression level, social support, satisfaction with life, and self-care ability of the *BMC*, respectively. The relationships between depression, social support, self-care, satisfaction with life, and adaptation were analyzed and a structural equation model was developed.

**Results:**

A total of 210 participants completed the questionnaire. The model demonstrated an acceptable fit of the data. The results showed that the difference between life satisfaction and depression on the level of adaptation was 60 and 23%, respectively. Social support and life satisfaction had a positive direct effect on the level of adaptation, both showing a positive correlation with the level of adaptation. Depression, on the other hand, have a direct effect on the level of adaptation and showed a negative correlation with the level of adaptation. Self-care ability indirectly influenced adaptation mediated by social support.

**Conclusion:**

Social support has a positive impact on both life satisfaction and depression, which in turn promotes adaptation. As a major source of social support, family members and nursing home staff in residential care facilities can enhance social support for older people through improved interaction, which can have a meaningful and positive impact on levels of adjustment. The model demonstrates the strengthening and weakening of social support, self-care, life satisfaction, and depression levels, which can help inform the development of relevant care health strategies for older people to promote levels of adjustment and improve quality of life.

## Introduction

The population of older people has been gradually increasing in China and abroad (1). In 2050, there are projected to be nearly 1.5 billion older adult persons in the world, up from the current 9.3% (2). It is challenging for other family members to care for the older adult people because of changes in family structure and the rise of special families (3). Family care is taken for granted in large families with multiple children, as promoted by traditional societies. However, the family structure has gradually been reduced to a nuclear family with a small number of children (4–7). In today’s society, women, who have traditionally been the primary family caregivers, are increasingly involved in social activities, leaving less time for family support tasks, which resulted in the older adult failing to accept excellent care at home (8). And On the premise that the new model of community pension has not yet been formed, the proportion of older adult people choosing institutional pension is gradually increasing (9, 10).

However, older people who unexpectedly leave their homes and enter residential care facilities in a new environment face a variety of maladjustment issues, including changes in psychological, physical, and social support, such as anxiety, despair, sadness, confusion, abandonment, and thoughts of suicide (11, 12). At the same time, they face loss of employment, isolation from family, friends, and community, the fragility of new relationships, loneliness, loss of privacy and identity, and breakdown of self-determination (13), and may even experience relocation trauma and relocation stress syndrome (14), which greatly affects the quality of life, mental health and quality of care of older people (15).

Adaptation is the process by which an older person moves into long-term care facilities, through perception, reflection, and conscious behavioral choices, accepts the nursing home as their home, reverses their sense of rejection in their new place of residence, develops a positive perception of life in the facility to achieve harmony between person and environment (8, 16). Life satisfaction refers to the positive sense of well-being experienced in adapting to life in a nursing home making the facility one’s home (17) and can be a potential indicator of successful aging and psychological adjustment (13). As older people enter residential care facilities, they were influenced by a number of factors in their adjustment to life in an institution including personal factors, family factors, nursing home factors, and social support system factors. Therefore, we propose in Hypothesis 1 that adherence to a combination of physical, psychological, social support, and life satisfaction factors associated with older adults would promote adaptation. Exploring the factors inherent in adaptation to provide a basis for developing specific intervention strategies is necessary to improve adaptation levels.

Social-ecological systems theory (SET) (18) deals with humans and their living environment and states that individual behavior is influenced by many internal and environmental factors, culminating in micro, meso, and macro ecosystems. The first layer is the micro system, which include the demographics, health status, and information awareness of individuals. Studies have shown that older people with planned admissions progress more quickly through the adjustment phase than those with unplanned admissions (19). And in the case of involuntary relocation, adjustment to the nursing home facility becomes very difficult and tends to increase mortality (8, 11). Secondly, increasing older people’s control over relocation decisions helps them to adapt better (20–22). Demographic characteristics include gender, age, education level, health level, number of children, and number of illnesses, which can all influence the state of older people’s adjustment (23). Chao et al. (9) showed that age, gender, education, and number of children were all factors that influenced the adaptation of older people. Thirdly, the health status of older people and their ability to perform activities of daily living were influenced by the level of adaptation of older people (8). Therefore, we propose in hypothesis 2 that positive psychology has a facilitative effect on the level of adaptation and negative emotions have a hindering effect on the level of adaptation. Secondly, good physical condition can influence adaptation levels directly and also indirectly through positive social support.

The next layer of the social-ecological system is the meso system, which consists of those systems directly related to the individual, such as family, community, organization, institution, etc. The meso system contains one or more environments that affect the “developing person.” Sun et al. (24) showed that life satisfaction with nursing home services, number of illnesses, length of stay, knowledge of the purpose of admission, resilience, and social support were all related to the level of adaptation of older people to long-term care life. Social support is primarily a combination of help from family, friends, and others to alleviate feelings of rejection and isolation caused by separation from family (25). These studies also found that social support from nursing home staff or other residents, the role of caregivers, and communication interactions were sufficient to influence the level of adaptation of older people (26–29). In previous studies, it was found that the management and services of residential care facilities influence the level of adjustment of the older adult and that the higher the level of satisfaction received by older people in the residential care facilities, the better the quality of care provided and the better the adjustment (30, 31). The highest layer of the social-ecological system is the macro system, which usually includes policies, culture, social norms, etc. Theoretically, the macro system influences human development through instantiated “coherence.” Three studies from Canada, the Philippines, and China (32–34) have demonstrated the impact of different cultural contexts on the adaptation of older adults, suggesting that beliefs can help them cognitively reorganize, understand, and give meaning to their new lives (35, 36). Therefore, we propose in Hypothesis 3 that social support can directly or indirectly influence the level of adaptation of older adults.

Internationally, most of the studies, such as Sun et al. (24), Koppitz et al. (37), Yoon et al. (30), Lane et al. (29), Sok et al. (8), Yu et al. (38) conducted in a cross-sectional manner have shown that physical, psychological, social system are influential factors in the adjustment of older adult people in residential care facilities. However, previous studies have not conducted systematic and comprehensive path analysis of adaptation influencing factors. Secondly, the researcher found that the Korean scholar Park et al. (39) modeled the life satisfaction of older adults in residential care facilities as an outcome indicator. In summary, researchers have not found path analysis studies of factors influencing the adaptation of older adults in nursing facilities. Therefore, it is necessary to study the structural causal relationship of older adult adjustment in residential care institutions under the combined influence of physical, emotional, social and psychological factors, life satisfaction and social support. In addition, international intervention strategies to promote adaptation among older adults in long-term residential care facilities are relatively lacking and one-sided (40–43). The objective of this study is to be able to provide a basis for the later development of intervention strategies that effectively improve the level of adaptation and enhance the quality of life of older people, and to provide reference suggestions for the development of policies related to healthy aging in other aging countries.

**Figure 1:**
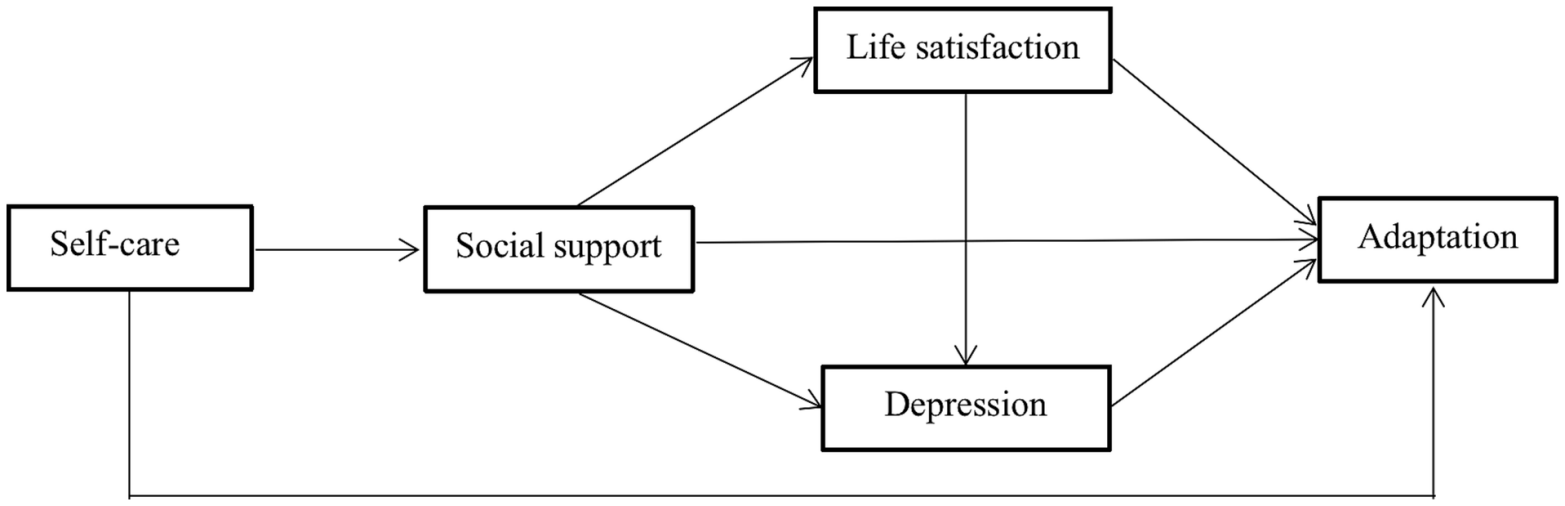
Constructed model for hypothesis testing.

In summary, the existing literature fails to address the causal relationships between physical, psychological and social factors of adaptation in older people in nursing facilities and reveals two problems. Firstly, there is little recognition of the importance of the relationship between these factors on the health outcomes of older people in residential care facilities. Secondly, the factors that influence the level of adjustment of older people in residential care facilities have been occasionally investigated without comprehensive theoretical guidance. Therefore, based on social-ecological systems theory and a review of relevant literature, this study aims to describe the structural causal relationships of adaptation in residential care facilities as influenced by a combination of physical, emotional, social and psychological factors, life satisfaction, and social support (44). Figure 1 shows the model we constructed for hypothesis testing.

## Methods

### Study setting and participants

A total of 220questionnaire-based survey was conducted from May 3 and August 31, 2022, out of which 10 were eliminated due to missing information, inaccurate records or implausible responses, etc. The final sample for analysis includes 210 individuals. This study used data from different older adult care institutions in Zhengzhou, China. These institutions, located in the eastern, western and northern regions, contribute to a fuller understanding and accurate identification of linkages between influencing factors. In addition, these institutions have more than 1,500 permanent residents. All interviewers were intensively trained prior to survey implementation. Participant recruitment eligibility criteria were: (a) consistented with the definition of older adults in Chinese law, only respondents aged ≥60 years were included; (b) abled to communicate verbally; (c) lived in a nursing home for more than 1 month; and (d) enabled to cooperate throughout the survey process. Exclusion criteria were: (a) those with impaired consciousness and severe speech and hearing communication impairments; and (b) those with other serious life-threatening comorbidities. The Life Sciences Ethics Review Committee from Zhengzhou University approved the study. Participants were informed of the importance of all aspects of this research investigation. Written consent was obtained after confirming their willingness to participate.

### Measures

#### Sociodemographic questionnaire

The participants’ general characteristics, including age, gender, marital status, duration of institutionalization, number of chronic physical illnesses, willingness to move in, source of funding, number of children, and readiness, were self-reported by the participants based on the questionnaire (24, 39, 45, 46).

#### Chinese Nursing Home Adjustment Scale (NHAS)

This is a 23-item self-assessment scale. The scale measures the adaptive level of the older adult in nursing home. The scale includes five dimensions: emotional distress, family feelings, relationship development, acceptance of new residence, and depressed mood. The items in the scale refer to the current five-point Likert type. Scores on the scale range from 23 to 115, with high scores indicating a high level of adaptation and good adjustment (47–49).

#### Geriatric Depression Scale-15 (GDS-15)

The standard version of the GDS-30 was simplified by Sheikh et al. (50) to be briefer and easier to assess. The scale measures depression levels in older adults. It contains 15 items, each of which is a question measuring the feelings of the older person over the last 1 week, with “yes” and “no” options. Each question has a score of 1 for “yes” and 0 for “no,” with a total score of 0 to 15. The higher the score on the scale, the more significant the depressive state, and a total score of ≥8 can be judged as having depressive symptoms, with a Cronbach’s alpha coefficient of 0.793 and retest reliability of 0.728 (51).

#### Social Support Scale (SSRS)

The scale is a social support scale that measures the level of social support in older adults. The scale has 10 entries and 3 dimensions, divided into objective support (3 items), subjective support (4 items), and support utilization (3 items). The total score is the sum of the scores of all 10 entries. Each item is scored from lowest to highest, with a total score of 40, the higher the score, the higher the level of social support, with Cronbach’s alpha coefficient of 0.80 and retest reliability of 0.92 (52–54).

#### Barthel Index

This scale is a self-care ability scale that measures the degree of self-care ability of the older adult. There are 10 items on the scale, with scores ranging from 0, 5, 10, and 15 for totally needing help to not needing help at all, and 5 or 10 for some items not needing help at all, for a total score of 100. The higher the score, the better the degree of ability to perform daily activities. The scores are divided into four groups: ≤40 for severe dysfunction, 41–60 for moderate dysfunction, 61–99 for mild dysfunction, and 100 for complete self-care. The Cronbach’s alpha coefficient of the scale was 0.92 and the half reliability was 0.87 (55).

#### Satisfaction with Life Scale (SWLS)

The Satisfaction with Life Scale (SWLS) was developed by Pavot scholars (56). This scale was used to measure the satisfaction level of life satisfaction of participants. The scale contains a total of 5 entries. Each item was rated on a 7-point Likert scale (1 to 7 representing “strongly disagree” to “strongly agree”) with a score range of 5 to 35, and the higher the respondent’s score, the higher the satisfaction with life. Cronbach’s α was 0.726 (57–59).

### Data collection

The data collection period for this survey was from May to August 2022. The study population was randomly sampled using a non-probability sampling method of older people aged 60 years and over. The data were collected in principle by means of a self-assessment questionnaire. When participants had difficulties filling in the questionnaire, the researchers helped them read the questions and write down the answers to the topics. Only 10 participants had difficulty filling in the questionnaire. After explaining the purpose of the study to the participants, the SPMSQ was used to measure the participants’ cognitive abilities and to determine the general characteristics of each participant. Questionnaires were then administered on their level of adjustment level, social support, life satisfaction, and depression.

### Data analysis

Descriptive statistics were presented as frequency (percentage) or mean (standard deviation) as appropriate. Continuous variables were analyzed by one-way analysis of variance (*ANOVA*) and the *t*-test, and categorical variables were analyzed by the chi-square or Fisher’s exact tests as appropriate. Spearman correlation coefficient tests were conducted to test the associations between social support, Adaptation level, depression, and life satisfaction. The ordinary least squares model was used to analyze the factors affecting the adaptation level by multiple regression. The level of adaptation was taken as the dependent variable, and those with statistical differences in the univariate analysis were taken as independent variables. The results were considered statistically significant at *p* < 0.05. All of the above analyses were performed using IBM SPSS Statistics for Windows, version 26.0 (IBM Corp., Armonk, NY, United States).

### Path analysis

To investigate the relationship between the Adaptation level, social support, depression, self-care life satisfaction, and sociodemographic characteristics, a path analysis model was developed and tested using Amos 26.0 (Amos Development Corp, Meadville, PA, United States). The path analysis was used to explore the direct or indirect dependencies among a set of variables including the demographics, social support, depression, self-care, and life satisfaction characteristics. The goodness of fit for the final model was assessed with the chi-square test and goodness of fit indices, such as the RMSEA, standardized root means square residual (SRMR), goodness-of-fit index (GFI), adjusted goodness-of-fit index (AGFI), normed fit index (NFI), incremental fit index (IFI), Tucker Lewis index (TLI), and comparative fit index (CFI). The values for GFI, AGFI, NFI, IFI, TLI, and CFI range from 0 to 1, with values greater than 0.90 indicating a good fit. Conventionally, there is a good fit if the RMSEA and SRMR are less than 0.05.

## Results

### General characteristics of study participants

The general characteristics of the study participants are shown in Table 1. More females (60.0%) than males (40.0%) were among the study participants. 48.1% of the respondents were in the age group of 80–89 years. Regarding marital status, 42.4% of the respondents had a spouse, and 57.6% of the respondents were unmarried, widowed, or divorced. The person who usually provides the most support to an older adult living in a senior care facility is usually a family member. Most of the older adults were voluntarily admitted to an institution (60.5%). 74.3% had chronic physical illnesses.

**Table 1:** General characteristics of study participants (*N* = 210).

Characteristic	Total (*N* = 210)	F/t/χ^2^	*P*
Sex			
Male	84 (40.0)	1.618	0.107
Female	126 (60.0)		
Age (years)			
60–69	13 (6.2)	1.343	0.261
70–79	55 (26.2)		
80–89	101 (48.1)		
≥90	41 (19.5)		
Length of stay at the institution (months)			
1–2	54 (25.7)	13,728	<0.001
3–5	55 (26.2)		
6–12	47 (22.4)		
≥13	54 (25.7)		
Whether to stay voluntarily			
Yes	127 (60.5)	0.448	<0.001
No	83 (39.5)		
Readiness			
Yes	103 (49.0)	10.454	<0.001
No	107 (51.0)		
Number of children			
≤2	50 (23.8)	0.314	0.815
3–5	89 (42.4)		
≥6	71 (33.8)		
Number of family visits			
1–3/weekly	16 (7.6)	5.053	0.001
4–6/weekly	56 (26.7)		
1–2/months	88 (41.9)		
1–2/years	41 (19.5)		
0	9 (4.3)		
Education level			
Uneducated	50 (23.8)	2.138	0.077
Elementary school	55 (26.2)		
Junior high school	44 (21.0)		
High school or junior college	40 (19.0)		
College and above	21 (10.0)		
Marital status			
No spouse	121 (57.6)	0.394	0.186
With spouse	89 (42.4)		
Number of chronic diseases			
0	54 (25.7)	7.708	0.001
1–3	86 (41.0)		
≥4	70 (33.3)		
Previous occupation			
Workers (farmers)	64 (30.5)	2.604	0.054
Unit personnel	84 (40.0)		
Business services/self-employed	24 (11.4)		
Unemployed person	38 (18.1)		
Self-care			
Severe dysfunction	43 (20.5)	0.751	0.523
Moderate dysfunction	79 (37.6)		
Mild functional impairment	62 (29.5)		
Fully self-care	66 (12.4)		
SSRS, Mean (SD)	27.41 ± 4.89	19.510	<0.001
GDS, Mean (SD)	7.86 ± 4.42	37.796	<0.001
SWLS, Mean (SD)	22.71 ± 6.84	32.910	<0.001

### Correlation analysis and multiple regression analysis

As shown in Table 2, we found statistically significant correlations (*p* all <0.01) between social support, depression, life satisfaction and level of adjustment. Social support, life satisfaction and self-care showed positive correlations with the level of adaptation, and depression showed a negative correlation with the level of adaptation. Of these, life satisfaction had the strongest positive correlation with total adaptation level. The range of the r coefficient was from 0.345 (relationship between self-care and level of adjustment) to 0.802 (relationship between life satisfaction and level of adjustment), indicating overall a weak (0.10–0.39) to strong correlation (0.70–0.89) (60). The results of the multiple regression are shown in Table 3. Length of institutional stay, social support, life satisfaction, self-care and depression were found to have an effect on the level of adjustment.

**Table 2:** Correlations between adaptation level, social support level, depression and life satisfaction (*N* = 210).

Item	Adaptation level	
	*r*	*P*
GDS	−0.776	<0.001
SWLS	0.893	<0.001
Self-care	0.345	<0.001
SSRS	0.760	<0.001
Length of stay at the institution (months)	0.435	<0.001

**Table 3:** Multiple regression analysis for factors predicting adaptation level in older adult adults in nursing home.

Variable	*B*	Standard error	*β*	*t*	*P*	95% CI	
						Low	High
Constant	53.628	3.356		15.979	<0.001	47.010	60.245
Length of stay at the institution (months)	0.638	0.278	0.066	2.292	0.023	0.089	1.187
SSRS	0.423	0.087	0.189	4.867	<0.001	0.251	0.594
GDS	−0.934	0.180	−0.207	−5.187	<0.001	−1.288	−0.579
SWLS	0.929	0.067	0.582	13.858	<0.001	0.797	1.062

SSRS, Social support; GDS, Depression; SWLS, Life satisfaction; *B* = Unstandardised coefficient; *β* = Standardized coefficient.

### Path analysis

Based on the results of the regression model, a path analysis model was developed to explore the relationship between social support, depression, self-care, life satisfaction, and adaptation. There are four representations in this model: (i) “depression” has a direct effect on adaptation (1.018); (ii) “life satisfaction” has a direct effect on adaptation (0.949); (iii) “social support” had a direct effect on adaptation (0.753); and (iv) “social support” mediated the relationship between “self-care” and “adaptation.”

**Figure 2:**
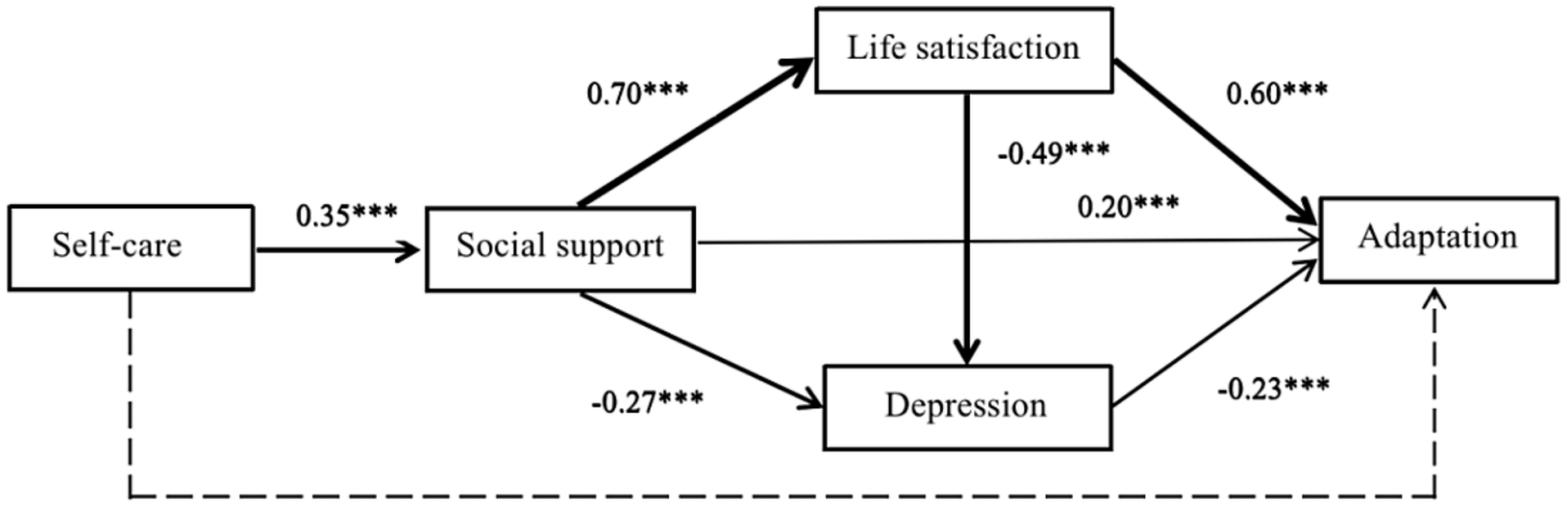
A structured path model of social support, self-care ability, depression, life satisfaction and adjustment of older adult people in Chinese nursing care facilities. ***P<0.001.

A structured path model of social support, self-care ability, depression, life satisfaction and adjustment of older adult people in Chinese nursing care facilities is presented in Figure 2, the model fits satisfactorily (*χ*^2^ = 2.338, *χ*^2^/df = 1.169, *p* = 0.311, RMSEA = 0.028, SRMR = 0.000, GFI = 0.996, AGFI = 0.967, CFI = 1.000, NFI = 0.997, IFI = 1.000, TLI = 0.998). The direct, indirect and total effects of the model are shown in Table 4.

**Table 4:** Standardized direct, indirect, and total effects for the final model.

Exogenous variables	Endogenous variables	SDE †	SIE ‡¶	STE §¶
Self-care	Adaptation		0.285***	0.285***
Social support		0.197***	0.556***	0.753***
Depression		−0.225***		−0.225***
Life satisfaction		0.595***	0.112***	0.707***

† SDE, Standardized direct effect; ‡ SIE, Standardized indirect effect; § STE, Standardized total effect; ¶ SMC, Squared multiple correlation; *p < 0.05, **p < 0.01, ***p < 0.001.

## Discussion

As the world’s population ages, the promotion and improvement of adaptation and quality of life of older adults in residential care facilities has been an important issue. In existing studies in the field of public health, research has shown that psychological status, social support, physical activity, and sociodemographic characteristics can influence the level of adjustment of older adults in long-term residential care facilities. However, these studies have focused on the segment- and discipline-specific issues and the one-way relationship between these factors and adjustment. Although prior research has identified an effect of these factors on adaptation (24, 26, 30, 31), it is unclear whether this effect is direct or indirect. By combining these factors into a more comprehensive framework in the study, we explored the relationships between sociodemographic, social support, psych-emotional, life satisfaction, and adaptation. And this is the first study to attempt to understand the path analysis of factors associated with the level of adaptation of Chinese older people relocating to nursing homes. Our study found an average level of adaptation among older people in residential care facilities, possibly due to the perception of Chinese cultural background and the presence of certain ideological burdens, which is in line with the findings of Chen et al. (61). Furthermore, our results suggest that social support, life satisfaction, and depression can, directly and indirectly, influence the level of adaptation in older adults. However, self-care did not have a direct effect on adaptation. This finding moves the field of adaptation forward by providing insight into the mediating role of social support in the relationship between self-care and adaptation.

According to previous studies, satisfaction, health status, length of stay, resilience, and social support are usually similar predictors (8, 10, 62, 63). These results confirmed the available evidence of disease co-morbidity and knowledge of the purpose of admission as key points for interventions. In terms of sociodemographic characteristics, our findings suggest that the level of adaptation of older adults may change depending on the length of stay and over time. The longer the length of long-term care residential facilities for older adults, the more likely they are to achieve high levels of adaptation, contributing to an improved quality of life. This is consistent with the existing literature (24), which suggests a positive relationship between the length of institutionalization and the level of adaptation (24). A longer length of stay in residential care facilitates better life adjustment for residents, but adaptation may be an ongoing dynamic process rather than a static state after stabilization (38). Brooke’s (35) reported that the disruption phase lasted 6–8 weeks after relocation. Therefore, interventions for older adults who have lived in the nursing home for less than a year can enhance the level of adjustment (10, 38).

In the path analysis, we already identified four variables (social support, life satisfaction, depression, and self-care) that were the strongest predictors of the level of adjustment in older people, similar to the results of Park et al. (39). The results suggested that changes in life satisfaction, social support, and depression may alter the level of adjustment by approximately 59%. This is similar to the findings of Jacelon et al. (64) where life satisfaction increased the level of adjustment in older adults. That is, the more social support older people felt, the better their satisfaction with life and the higher their level of adjustment (65, 66). This is similar to the findings of Lee et al. (67). In addition, mainstream Chinese culture is deeply rooted in Confucianism, and filial piety is a core virtue of society (68). Therefore, most residential care facilities promote the creation of a caring culture with filial piety and a home-like atmosphere to help older adults gain a sense of belonging. Fitzpatrick et al. (63) reported the need for nursing home staff to understand organizational approaches and ethnic cultures to facilitate transitions and create a culture of care for older adults. This study found that depression levels were negatively associated with adaptation. In addition, depression can indirectly affect adaptation through social support. These findings suggest that the level of depression is a very important factor with special implications for promoting adaptation in older adults in nursing care facilities. Possible reasons for this are, on the one hand, that some older adults are not aware that their functional status has changed. On the other hand, their family members do not inform them or consult them and make decisions directly. This can cause older adults to feel abandoned and frustrated, to show resistance to their new environment, and to express dissatisfaction. This suggests that family members and nursing staff should understand the true purpose of their admission, which is the first step in improving the level of adaptation.

Furthermore, we found an indirect effect of self-care ability on the level of adaptation, with social support as a mediating factor. The higher the ability to take care of oneself in daily life, the higher the level of adaptation (8). Self-care ability plays a central role in the process of adapting to the nursing home. As older people’s physiological functions decline, their self-care ability becomes poorer and their need for healthcare increases, and residential care facilities are often unable to provide timely and effective healthcare services, leading to an increased burden on the minds of some older people and thus creating or exacerbating psychological adjustment problems.

In a study by Cui et al. (69), it was found that older people who are physically healthy and have good self-care skills have greater control over their lives, better psychological status, and more opportunities for social interaction. Social support is a variety of help from family, friends, and others. The more support older adults perceive, the better they adapt to life in a nursing home, which is consistent with previous research (62). Some studies (70, 71) by have shown that older adults’ relationships with family and friends influence the level of adaptation. As older people are separated from their families when they move into residential care facilities, they developed a sense of rejection and severe isolation (37). And families can provide emotional support and meet the spiritual comfort of the older adult. In addition, it is not easy for older adults to establish new social networks. Because of the age of the older adult around them, the cognitive impairment of some of them, and the busy work of the nursing home staff, this results in limited access to social support, which in turn leads to the idea of going home for the older adult (9). Social support is the second focus of the intervention strategy. Ciccone et al.’s study (72) reported that older adult people’s health and self-management abilities developed overwhelmingly positive outcomes through a strong “partnership” between the care manager and the patient, as well as through effective collaboration between the physician and the care manager. Therefore, facility staff should actively intervene and work with older people to provide better care for their illnesses, help them to improve their self-care skills, provide regular rehabilitation exercises and offer appropriate assistance to facilitate their adjustment to the nursing home environment. While at the same time, it is important to focus on the important role that social support plays in the process of adaptation (62), enabling them to develop and maintain good relationships with other residents and facility staff and to maintain family functioning. A variety of educational programs for facility staff must be developed to enhance the level of relationship-building skills and professionalism of staff.

With regard to the implications for clinical practice, first, the study has found that only depression presents a negative correlation to adaptation in older adults. This is crucial for health professionals. Professionals should pay more attention to the development of interventions to maintain and promote the level of adaptation of older adults moving to residential care facilities. Secondly, according to the path model in this study, considering the positive correlation of social support, interventions that increase social support are expected to increase the level of adjustment of older adults. For example, residential care facilities can foster higher levels of social support and enhance adjustment by organizing family activities that enhance frequent interactions and increase familiarity and bonding among peers. Thirdly, some physical characteristics, such as self-care, do not have a direct impact on adjustment. However, self-care ability plays a central role in the process of older adult people’s adaptation to residential care facilities. As the physiological functions of older adults decline, their self-care skills become less capable, and their need for health care increases. While most residential care facilities fail to provide timely and effective health care services, leading to an increased burden on the minds of some older adults, thus creating or exacerbating psychological adaptation problems. Therefore, nursing home staff should pay attention to the self-care capacity of older people and develop policies to enhance their self-efficacy, maintain their self-esteem and improve their life satisfaction. This is because it is a directly relevant factor that affects the level of adaptation of older adults and plays an important role in their health and quality of life. In addition, the overall goal of providing healthcare services to older people living in nursing homes is to optimize their health, well-being, and quality of life as a primary goal of active living. According to our findings, person-centered care is considered necessary to improve the level of adjustment of older people in nursing homes. Older people can bring familiar objects that create a sense of home in their rooms, maintain a sense of well-being and identity (73), and create continuity with their previous homes. Older people lose their previous social networks after admission to a nursing home (9, 74), while caregivers play an extremely important role in interpersonal relationships (66). Therefore, caregivers should maintain older people’s connections with family and friends and promote new connections with peers (34), enhance social interactions, build good interpersonal relationships, and enhance personal confidence and emotional resilience (75, 76). At the same time, researchers must focus on the important role that social support plays in the adaptation process (62) to enable older adults to develop and maintain good relationships with family and peers and to maintain family functioning. And it is recommended that researchers develop various educational programs for nursing home staff to improve professional skills and proficiency.

There are some limitations to this study. In terms of methodology, including: (1) First, this is a cross-sectional study. The relationship between these factors and the findings is exploratory in nature. Only quantitative findings were conducted in this study, which showed correlated factors. However, the explanation of these correlations needs to be complemented by qualitative research. (2) Three nursing homes of the study were selected by Henan Province China and a random sample was used. Therefore, there are limitations to generalizability. Researchers should recruit participants in different regions to confirm generalizability for future studies. (3) The data collected for this study were in principle based on a self-administered questionnaire with a large number of questions. Therefore, this is a limitation on the accurate representation of individuals. And the cross-sectional surveys limit the exploration of older adults’ adaptive capacity over time. (4) Since the configuration and structural framework of the nursing homes included in this study are similar, the number of nursing home staffs or the number of beds in the nursing institutions is not included in the structural model as an influencing factor. In terms of theory, including: (1) This study is limited in its application of this theoretical framework with respect to the lack of cultural context. (2) The theoretical framework is more widely used in ecological disciplines, and its application in medicine and other disciplines is in a period of development. (3) The theory has been applied by scholars to enrich intervention programs for childhood asthma or to explain the determinants of telemedicine, but its application in the context of residential care facilities is in a developmental period.

With regard to the suggestions for future research. (1) It is suggested that other researchers can apply the results of this study to populations such as autistic, migrant older adult, empty nesters, and mobile children. (2) Researchers could conduct longitudinal studies that employ larger sample sizes and broad sampling areas to further test the structural model and develop interventions and transition trajectories. (3) Our team suggests that researchers could also conduct experimental studies for practical and effective intervention development and application to improve the level of adaptation of older adults in residential care facilities. Secondly, a culture of care needs to be emphasized and the value of filial piety should be promoted. (4) A qualitative study should be conducted to gain insight into the inner world, needs, and life satisfaction of older adults in nursing care facilities, combining quantitative and qualitative research to better explore the influencing factors of adaptation. The explanation for these correlations can be complemented through qualitative research (77). (5) It is recommended that the management of residential care facilities should be standardized and monitored by the government. There is a need for care centers to develop more innovative models that focus on actively accompanying older adults in long-term residential care facilities in a comfortable manner. For example, through positive care practices, families and nursing home staff should be helped to become more aware of these positive ways of interaction, which in turn will continue to promote the adaptation of the older adult to the residential care facility. (6) This study provides practical insights for the future advancement of adaptation interventions for older adults in residential care facilities. (a) At the micro layer, health administration departments should pay more attention to residential care facilities, families, and older people’s proper awareness of relevant information and knowledge, and strengthen nursing home staff to conduct skills training to improve operational skills. (b) At the meso layer, residential care facilities should establish appropriate incentives to encourage the willingness of older people to get out of their rooms. At the same time, nursing homes should choose to implement internal resource allocation and create their own online healthcare platform to provide a useful online healthcare environment for older adults. Residential care facilities can advocate or organize internal activities to facilitate the exchange of experiences among older adults. (c) At the macro layer, the government should improve the laws and policies related to senior care. Meanwhile, mass media should be guided to actively report and publicize policies related to nursing homes.

## Conclusion

In summary, the appropriateness of a path model using multiple factors that influence the level of adaptation of older people in aged care facilities can explain their adaptation. This study found that social support, life satisfaction, self-care, and depression influence adaptation to living facilities for older people. Based on the findings of this study, it can be concluded that enhancing these factors that have been validated will lead to the successful adaptation of older adults living in residential care facilities. This study provides evidence for intervention factors to improve the level of adaptation of older adults in nursing homes.

## Data availability statement

The original contributions presented in the study are included in the article/supplementary material, further inquiries can be directed to the corresponding authors.

## Ethics statement

This study was approved by the Zhengzhou University Life Science Ethics Review Committee (ZZUIRB2021-16). The participants provided their written informed consent to participate in this study.

## Author contributions

DZ, PW, and YS: design of the manuscript. DZ, YXS, SFZ, and RM: acquisition, analysis, or interpretation of data. MlN, DZ, and LZ: drafting and substantively revising the work. DZ, PW, RM, and SFZ: constructive discussion. All authors have read and approved the manuscript.
